# Supplementary material for: The Ca2+-Sensitivity of Contraction is Increased in the Left Atrium and Left Ventricle of Patients with Ischemic Heart Failure
Source: bioRxiv. 2026 Jul 1:2026.06.26.734899. Preprint. [Version 1] doi: 10.64898/2026.06.26.734899 (PMC13345188; doi:10.64898/2026.06.26.734899)
Supplement: Supplement 1 [file media-1.pdf]

Supplementary Materials

The Ca^{2+}-sensitivity of contraction is increased in the left atrium and left ventricle of patients with ischemic heart failure

Gregory N. Milburn†[a,b], PhD, Chloe Roth†[a,b], BS, Jania Bell[b], BS, Austin Wellette Hunsucker[a,b], PhD, Marziyeh Pakbaz[b], MD, Alex Lewalle[c], PhD, Steven A. Niederer[c], DPhil, Kenneth S. Campbell[b], PhD

[a] Department of Physiology, University of Kentucky, Lexington, Kentucky, 40536, United States of America

[b] Division of Cardiovascular Medicine, University of Kentucky, Lexington, Kentucky, 40536, United States of America

[c] National Heart and Lung Institute, Imperial College London, London, UK

† Authors contributed equally to this work

Contents:

**Supplemental Material**	**Page**
Supplemental Table 1. Summary Group Characteristics	**2**
Supplemental Table 2. Individual Patient Characteristics	**3**
Supplemental Figure 1. Myocardial strip dimensions did not differ between ischemic and non-failing samples	**4**
Supplementary Figure 2. Left ventricle and atria have different force transients in response to step stretches	**5**
Supplementary Figure 3. Left ventricle cross-bridge kinetics are slower at longer sarcomere length	**6**
Supplementary Figure 4. Sarcomere length and disease status do not significantly alter left atrial cross-bridge kinetics	**7**

Supplemental Table 1. Summary Group Characteristics

	Donor (n = 10)	Ischemic HF (n = 10)	p-value
Age	52.3 ± 11.8	62.4 ± 9.8	0.053
BMI	26.5 ± 3.8	27.3 ± 3.7	0.63
HbA1C	5.4 ± 0.6	6.5 ± 2	0.14
LVEF (%)	55.4 ± 12.4	22.3 ± 5.1	< 0.001
LVIDd (cm)	4.5 ± 0.5	6.2 ± 0.8	< 0.001
LVPWd (cm)	1.1 ± 0.2	0.9 ± 0.1	0.064

Patient clinical characteristics and demographics. Values are reported as mean ± standard deviation. BMI = body mass index; HbA1C = hemoglobin A1C; LVEF = left ventricle ejection fraction; LVIDd = left ventricle interior diameters at diastole; LVPWd = left ventricle posterior wall thickness; HF = heart failure.

Supplemental Table 2. Individual Patient Characteristics

Patient ID	Patient Group	Race	Sex	Age	Donor cause of death	Diabetic	BMI	HbA1C	LVIDd (cm)	LVPWd (cm)	Ejection fraction	Coronary vessel(s) with disease
2508D	Donor	White	Male	41	CVA/Stroke	No	32.1	5.3	5.2	1.4	31	-
2B487	Donor	White	Male	42	TBI	No	31.0	5.3	4.1	0.99	55	-
2DDC0	Donor	White	Male	49	TBI	No	24.4	5.2	5	0.8	56	-
30B2B	Donor	White	Male	71	Anoxic brain injury	No	24.2	5.2	4	1.2	66	-
5245C	Donor	White	Male	55	CVA/Stroke	No	19.7	4.8				-
B23E3	Donor	White	Male	50	CVA/Stroke	No	29.4	5.5	4.2	0.99	55	-
BC90C	Donor	White	Female	39	Anoxic brain injury	No	24.1	5.2	4.4	0.98	55	-
BE318	Donor	White	Male	46	CVA/Stroke	Yes	24.5	7.0	4.7	1.1	70	-
8CB30	Donor	White	Female	61	TBI	No	28.7	-	-	-	-	-
A9200	Donor	Black or African American	Male	70	CVA/Stroke	Yes	26.5	5.4	-	-	-	-
0DC6E	Ischemic	Black or African American	Male	55	-	Yes	32.9	6.1	4.9	1.1	32.9	LAD, RCA, PDA
2256F	Ischemic	Black or African American	Male	64.26	-	No	25.7	5.7	6.7	1.1	14.8	LAD, PDA
2D65C	Ischemic	White	Male	63	-	Yes	25.6	8.5	6.3	0.78	21.9	LAD,RCA, Cx
5845F	Ischemic	White	Male	71	-	No	30.6	6.0	6.7	1	20	LAD
9664E	Ischemic	White	Male	42	-	No	27.4	5.5	5.8	0.75	21	LAD,RCA
AF1FF	Ischemic	White	Male	75	-	No	32.8	5.2	7.1	0.83	23	LAD
D6A67	Ischemic	White	Male	53	-	No	23.0	5.6	6.4	0.77	20	LAD,RCA, Cx
DFEFA	Ischemic	White	Male	67	-	No	22.7	5.3	-	-	-	LAD,RCA, Cx
FAC13	Ischemic	White	Male	65	-	No	25.5	5.8	7.1	0.79	20	LAD
FCFAD	Ischemic	White	Male	69	-	Yes	26.5	11.7	5.2	0.96	27	LAD,RCA

CVA = cerebrovascular attach; TBI = traumatic brain injury; BMI = Body mass index; HbA1C = hemoglobin A1C; LVIDd = left ventricle interior diameter at diastole; LVPWd = left ventricle posterior wall thickness; LAD = left anterior descending; RCA = right coronary artery; PDA = posterior descending artery; Cx = circumflex

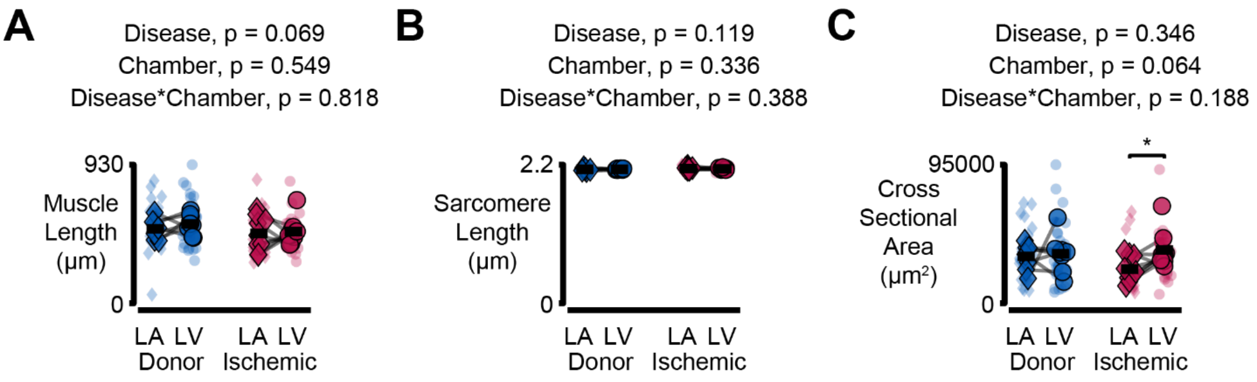

Supplemental Figure 1. Myocardial strip dimensions did not differ between ischemic and non-failing samples

Superplots comparing multicellular myocardial strip (A) length, (B) sarcomere length, and (C) cross-sectional area between ischemic heart failure and non-failing samples at a sarcomere length of 2.2 microns. Transparent symbols represent individual muscle preparations, while opaque symbols represent the mean of each patient. Data were analyzed using linear mixed models. *$p < 0.05$, **$p < 0.01$, ***$p<0.001$

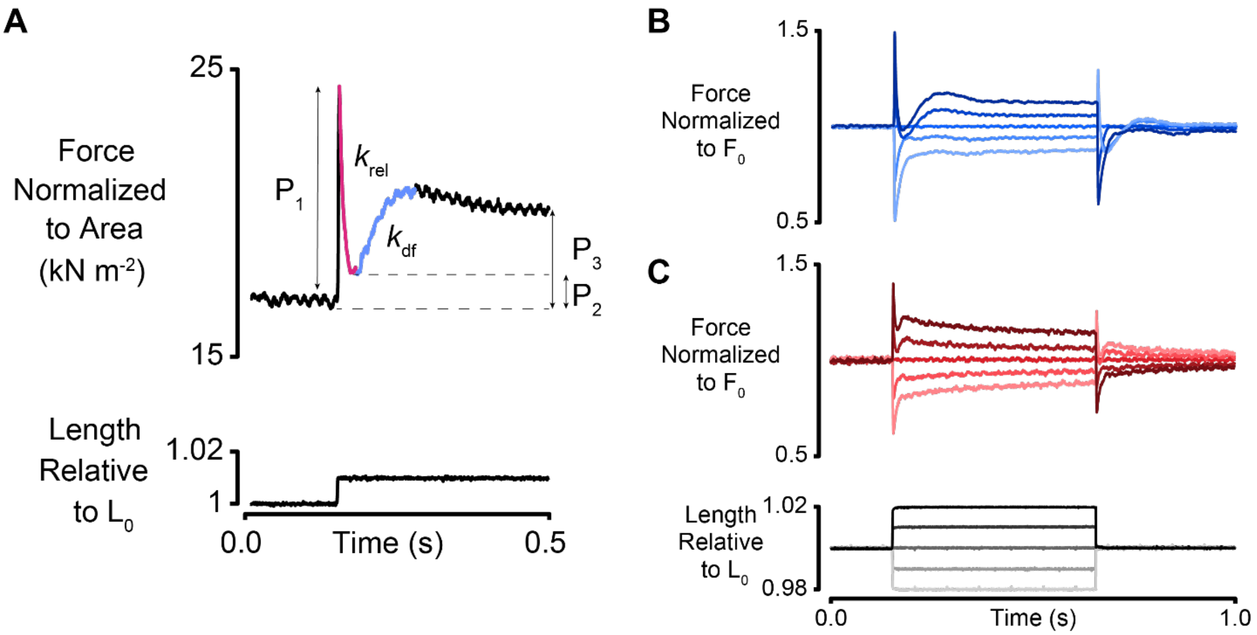

Supplementary Figure 2. Left ventricle and atria have different force transients in response to step stretches

To examine cross-bridge dynamics, (A) preparations at steady state force were rapidly lengthened 1% of the initial muscle length (L_0) and the resulting force transient recorded. The force transient in response to step length changes differs between (B) left ventricle and (C) left atria myocardium.

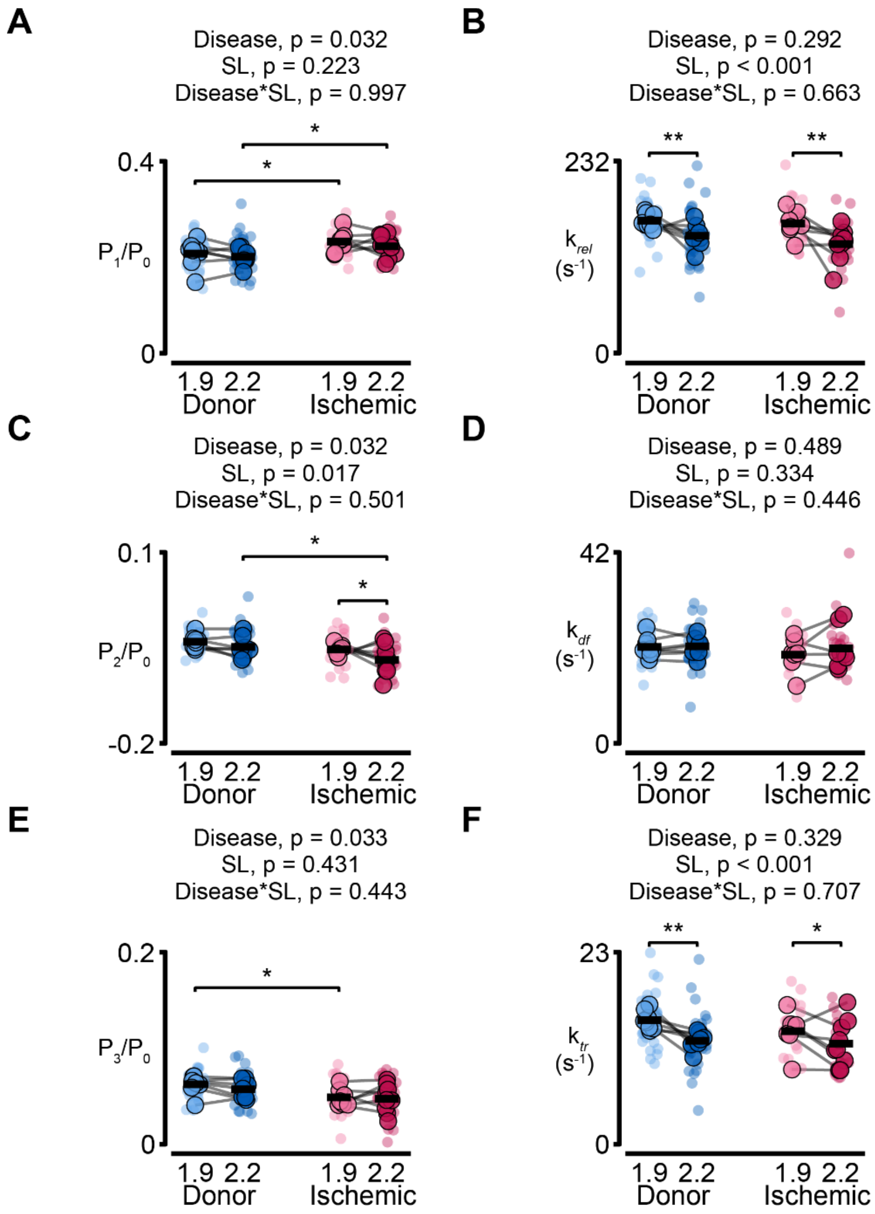

Supplementary Figure 3. Left ventricle cross-bridge kinetics are slower at longer sarcomere length

Parameters calculated from step-stretch force transients or ktr maneuver. Superplots show (A) P1, (C) P2, and (E) P3 normalized to P0 and (B) k_{rel}, (D) k_{df}, (F) k_{tr}. Data were analyzed using linear mixed models. *$p < 0.05$, **$p < 0.01$, ***$p<0.001$

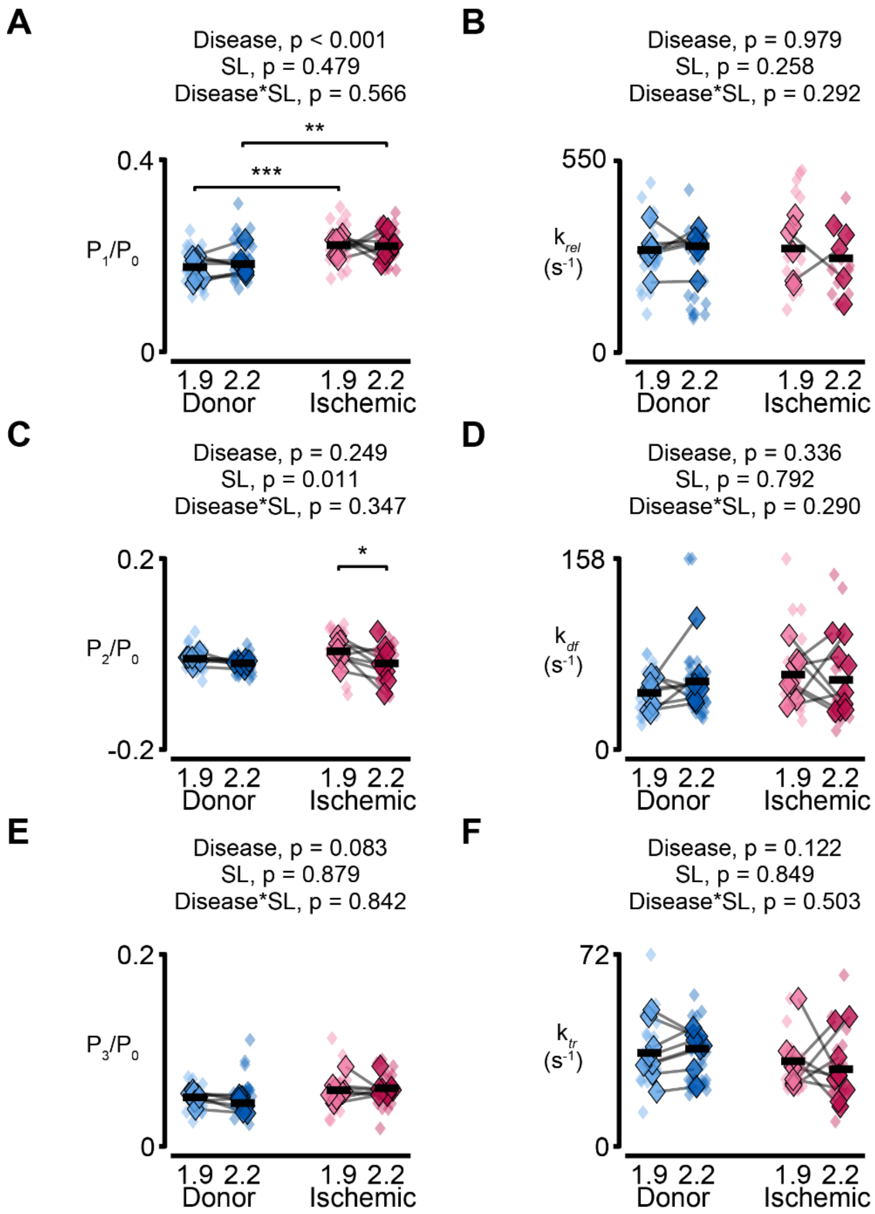

Supplementary Figure 4. Sarcomere length and disease status do not significantly alter left atrial cross-bridge kinetics

Parameters calculated from step-stretch force transients or ktr maneuver. Superplots show (A) P1, (C) P2, and (E) P3 normalized to P0 and (B) k_{rel}, (D) k_{df}, (F) k_{tr}. Data were analyzed using linear mixed models. *p < 0.05, **p < 0.01, ***p<0.001
